# Lipid and Nucleocapsid N-Protein Accumulation in COVID-19 Patient Lung and Infected Cells

**DOI:** 10.1128/spectrum.01271-21

**Published:** 2022-02-16

**Authors:** Anita E. Grootemaat, Sanne van der Niet, Edwin R. Scholl, Eva Roos, Bernadette Schurink, Marianna Bugiani, Sara E. Miller, Per Larsen, Jeannette Pankras, Eric A. Reits, Nicole N. van der Wel

**Affiliations:** a Electron Microscopy Centre Amsterdam, Medical Biology, Amsterdam University Medical Centre (UMC), Amsterdam, the Netherlands; b Department of Pathology, Amsterdam University Medical Centers (UMC), VU University Amsterdam, Amsterdam, the Netherlands; c Department of Pathology, Duke University Medical Center, Durham, North Carolina, USA; Texas A&M University

**Keywords:** COVID-19, pathology, electron microscopy, lipids

## Abstract

The pandemic of the severe acute respiratory syndrome coronavirus 2 (SARS-CoV-2) has caused a global outbreak and prompted an enormous research effort. Still, the subcellular localization of the coronavirus in lungs of COVID-19 patients is not well understood. Here, the localization of the SARS-CoV-2 proteins is studied in postmortem lung material of COVID-19 patients and in SARS-CoV-2-infected Vero cells, processed identically. Correlative light and electron microscopy on semithick cryo-sections demonstrated induction of electron-lucent, lipid-filled compartments after SARS-CoV-2 infection in both lung and cell cultures. In lung tissue, the nonstructural protein 4 and the stable nucleocapsid N-protein were detected on these novel lipid-filled compartments. The induction of such lipid-filled compartments and the localization of the viral proteins in lung of patients with fatal COVID-19 may explain the extensive inflammatory response and provide a new hallmark for SARS-CoV-2 infection at the final, fatal stage of infection.

**IMPORTANCE** Visualization of the subcellular localization of SARS-CoV-2 proteins in lung patient material of COVID-19 patients is important for the understanding of this new virus. We detected viral proteins in the context of the ultrastructure of infected cells and tissues and discovered that some viral proteins accumulate in novel, lipid-filled compartments. These structures are induced in Vero cells but, more importantly, also in lung of patients with COVID-19. We have characterized these lipid-filled compartments and determined that this is a novel, virus-induced structure. Immunogold labeling demonstrated that cellular markers, such as CD63 and lipid droplet marker PLIN-2, are absent. Colocalization of lipid-filled compartments with the stable N-protein and nonstructural protein 4 in lung of the last stages of COVID-19 indicates that these compartments play a key role in the devastating immune response that SARS-CoV-2 infections provoke.

## INTRODUCTION

The outbreak of severe acute respiratory syndrome coronavirus 2 (SARS-CoV-2) in late 2019 is the third major outbreak of β-coronaviruses in the human population of the past two decennia, together with the smaller outbreaks of severe acute respiratory syndrome coronavirus (SARS-CoV-1) in 2003 and Middle East respiratory syndrome coronavirus (MERS-CoV) in 2012.

SARS-CoV-2 belongs to the family *Coronaviridae*, a large family of single-stranded positive-sense RNA [(+)RNA] viruses. The genome codes for a number of polyproteins that, once processed by proteases, produce nonstructural proteins involved in viral replication (1). In addition, four structural proteins are produced: envelope (E), membrane (M), nucleocapsid (N), and spike (S). Coronaviruses are well known for their ability to induce high membrane plasticity in host cells, where the membrane rearrangements lead to the formation of viral replication organelles (ROs) (2–10). As observed in SARS-CoV-1, MERS-CoV, and the closely related coronavirus murine hepatitis virus (MHV), the ROs consist of convoluted membranes (CMs) that are interconnected with double-membrane vesicles (DMVs) and appear to be continuous with the membranes that constitute the endoplasmic reticulum (ER) (2, 11–17). Elaborate studies using immunofluorescence and electron microscopy (EM) techniques demonstrate that DMVs contain double-stranded RNA (dsRNA), which can be used as a marker of (+)RNA virus replication (2, 3, 18, 19). Taken together, these findings indicate that the RO serves as the replication and transcription site in which the DMVs may provide a zone safe from detection by the innate immune sensors and degradation by RNA degradation machinery in the host cell (20, 21).

The formation of DMVs has been shown to be facilitated by coronaviral nonstructural proteins (nsps) (22). Coexpression of three virally encoded transmembrane proteins, namely, nsp3, nsp4, and nsp6, has been found to be sufficient for the production of DMVs in SARS-CoV-1 and MERS-CoV, where the interactions of nsp3 and nsp4 result in the pairing and curving of membranes and nsp6 contributes to the production of vesicles (9, 10, 23). A recent publication, using cryo-electron tomography (cryo-ET), shows DMVs of SARS-CoV-2 and MHV in a native host cellular environment containing pore complexes that were not found in previous studies using conventional EM methods (18). Additionally, the publications by Wolff et al. (18) and Klein et al. (17) demonstrate the presence of N-protein in these DMVs.

The subcellular localization of the viral proteins and virus particles is based on infections in cultured cells. In patient material, viral proteins have been localized at a cellular level in various organs of COVID-19 patients (24), including human kidney (25), and in lungs of cynomolgus macaques (26). These studies used light microscopy to find regions of interest, and some of the studies subsequently used EM to find virus particles. One of the hurdles to overcome is the correct identification of viral particles in patient material, such as lung (12, 27–31), kidney (32–38), and other organs reviewed in reference 6. Recent publications show data on the morphology and size of isolated SARS-CoV-2 particles (39–42) and virus particles in Vero E6 cells (17) with the use of conventional EM and cryo-EM, although these data alone are not always sufficient to recognize viral proteins or virus particles. Bullock et al. proposed a set of eight rules for the correct identification of coronaviruses (6). Following these rules, a closer inspection of 27 articles where supposed SARS-CoV-2 particles in patient-derived samples have been found revealed that, according to Bullock et al., only four articles correctly identified virus (6, 43–46). The most common misinterpretations were clathrin-coated vesicles as single SARS-CoV-2 particles and endosome-derived multivesicular bodies (MVBs) as ROs (6, 46).

To assist in this identification conundrum, labeling of antibodies directed against specific viral proteins can be of use. In this article, we provide the first insights into the localizations of both structural and nonstructural proteins in SARS-CoV-2-infected Vero cells and compare these with identically processed patient samples retrieved during the first wave of SARS-CoV-2 infections using immunogold labeling and correlative light-electron microscopy (CLEM).

## RESULTS

### Immuno-electron microscopy on SARS-CoV-2-infected Vero cells.

Since the outbreak of COVID-19, the identification of virus particles using EM in lung has been a heavily debated subject (6, 47, 48). Based on the morphology, it is, especially in postmortem material, difficult to discriminate single virus particles from clathrin-coated vesicles, and MVBs have been interpreted as clusters of virus particles. Therefore, we decided to employ immunogold labeling, which can be used to decorate (viral) proteins specifically with 10- or 15-nm gold particles to distinguish them from cell organelles. This way, virus particles with M-, N-, or S-protein and the replication complexes with nonstructural proteins can be identified by the gold attached to the specific antibodies. To validate whether the antibodies used for recognition of the proteins in florescence microscopy (FM) (49) can be used on patient materials fixed with an extended fixation protocol, we first tested these antibodies on SARS-CoV-2-infected Vero cells. The antibodies were used on uninfected and 24-h-infected Vero cells fixed for 1, 3, and 14 days, as we had fixed patient material in a similar manner. Different antibodies against viral proteins were tested (see Materials and Methods), and successful labeling and their subcellular localizations are described.

### Characterization of virus particles with N-protein.

**FIG 1:**
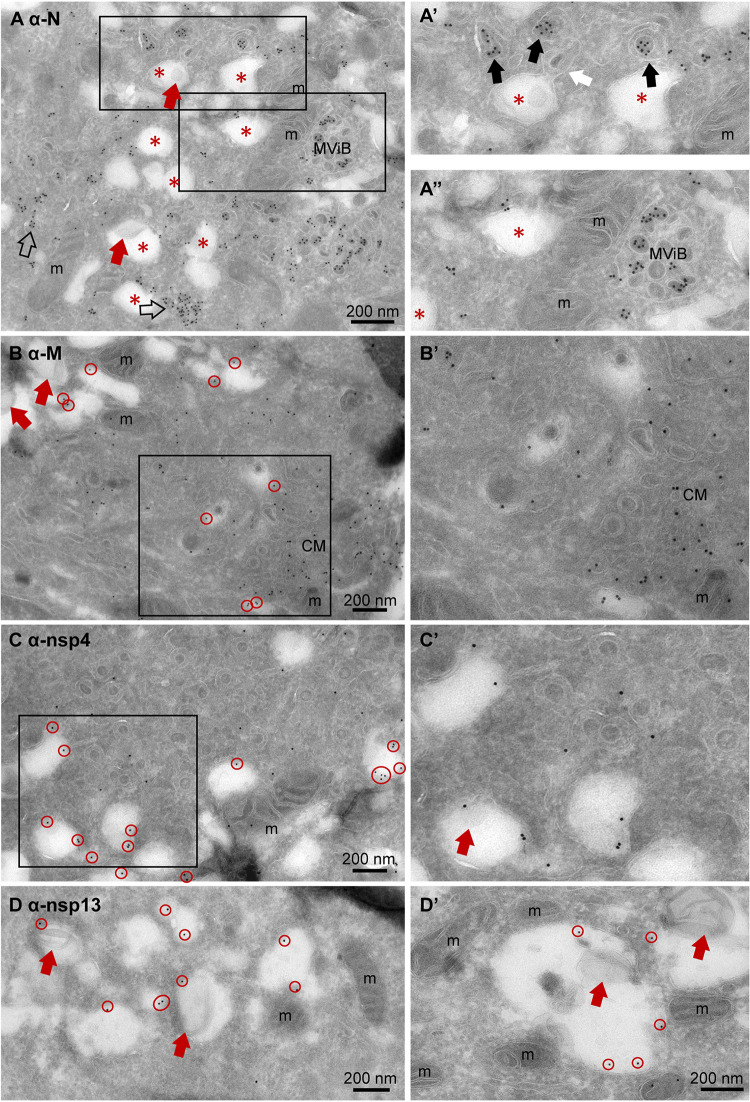
Subcellular localization of viral proteins in infected Vero cells. Vero cells were infected with SARS-CoV-2 for 24 h and immuno-EM labeled with antibodies against SARS-CoV-1 proteins, followed by secondary antibodies conjugated to 10-nm gold particles. (A) Clusters of N-protein labeling in cytosol (open arrows) and (enlarged in A’) on double-membrane spherules (right-most black arrow), or virus particles enclosed in a single membrane (two left-most black arrows). From the e-lucent compartment (red *), a “virus-like” particle (as it is without N-protein labeling) is budding (white arrow). (A’’) Enlarged and not enlarged area with MViB containing labeled and unlabeled virus-like particles. (B) M-protein immunogold labeling on e-lucent compartments (gold is circled in red); in enlarged box, immunogold labeling on convoluted membrane structure (CM). Note virus-like particles are not labeled. (C) Immunogold labeling of nsp4 on e-lucent compartments (circled in red) and various virus-like particles enclosed in a membrane without nsp4 labeling, also enlarged in panel C’. (D) Immunolabeling of nsp13 on e-lucent compartments containing lipid-like structures (red arrows). (D’) Higher magnification of panel D. Immunogold decoration on e-lucent compartments is indicated by red circles, mitochondria by m, multiple virus body by MViB, convoluted membrane structure by CM, lipid-like structures by red arrows, N-protein in cytosol by open arrows, N-protein labeled virus by black arrows, and enlarged area by black boxes.

Immunogold labeling of SARS-CoV-1 structural proteins using a mouse anti-SARS-CoV-1-N (46-4) antibody demonstrated that the nucleocapsid protein (N-protein or N) is detected in the cytosol and on virus particles in several subcellular structures (Fig. 1, Fig. S1) of infected cells. The N-protein can be detected specifically, as no labeling was detected on uninfected cells. Therefore, all N-protein-positive, membrane-enclosed spherical structures ranging in size from 60 to 120 nm in diameter and with an electron-dense core (e-dense, black) (6, 40) are annotated here as virus particles. Note that in cells and tissues stained with osmium and embedded in resin, membranes appear e-dense, whereas using the immuno-EM method on cryo-sections, membranes appear electron-lucent (e-lucent, white) (50). This is due to the fact that with the immuno-EM method, membranes are not stained but only surrounding proteins in the cytosol are stained with uranyl acetate. In 24-h-infected Vero cells, small clusters of N-protein can be detected in proximity to double-membrane structures, bending around the N-protein cluster similarly to those in the cryo-EM sections (Fig. S1) (18). Coronaviruses are known to be membrane-enveloped viruses, detected mostly inside host membrane structures (6, 51), and indeed the majority of the virus particles are surrounded by membranes (3, 17) (Fig. 1A and B). Intact viruses are also identified close to the Golgi (Fig. S2), inside multivirus bodies (MViB) (Fig. 1A, Fig S1, Fig. S3), and inside open e-lucent structures (Fig. S4). Both spherical and oval-shaped virus particles are visible. The sizes of the virus particles are measured inside MViBs and in the cytoplasm and are categorized as spherical or oval-shaped. All particles are measured at the longest axis of N-protein-positive particles that have a clear membrane and e-dense core present. The average size between the spherical and oval-shaped virus is slightly different but not statistically significant. Inside MViBs and in the cytoplasm, spherical particles are identical: 87 nm ± 17 nm versus 108 ± 27 nm and 113 ± 28 nm for the oval-shaped particles (Table 1). Different EM techniques result in slightly different sizes, being 97 ± 12 nm for oval-shaped cryo-EM fixed extracellular SARS-CoV-2 (42) or 99 nm in resin-embedded spherical virus (40). Thus, in 24-h-infected Vero cells, N-protein-positive virus particles can be detected as spherical 87-nm to 113-nm oval-shaped membrane structures with an e-dense core, present in the cytosol, close to Golgi, or in MViBs.

**TABLE 1:** Average particle size at different subcellular locations^*a*^

Subcellular location	Data for particles in subcellular location:			
	MViB		Cytoplasmic	
Virus shape	Spherical	Oval	Spherical	Oval
*x* in nm	87 ± 17	108 ± 27	87 ± 17	113 ± 28
*n*	61	21	59	8

a Average size of virus particles in multivirus bodies (MViB) and in cytoplasm was measured and presented as average size (*x*) ± standard deviation, and number of virus particles was measured (*n*) in Vero cells infected with SARS-CoV-2 for 24 h and immunogold labeled for N-protein with 10-nm gold.

### Classification of virus-containing compartments.

As the presence of SARS-CoV-2 in multivesicular structures in lung is heavily debated (6, 47), we studied the presence of lysosomal markers like CD63 in the multivirus bodies we showed to be N-protein positive (Fig. 1, Fig. S1 and S3). The Vero cell line is a kidney epithelial cell line from African green monkey, but antibodies against human CD63, a glycosylated transmembrane protein containing a putative lysosomal-targeting/internalization motif, can be detected in multilamellar bodies (MLB), which are lysosomal compartments. Only some CD63 label is detected in the multivesicular bodies (Fig. S3F). Therefore, we propose that the compartments in which the viral N-protein is detected are not true lysosomes but, rather, multivirus bodies. More elaborate studies on different stages of infection and blocking lysosomal acidification combined with immuno-EM have to be performed to determine the role of these MViBs during viral replication.

**FIG 2:**
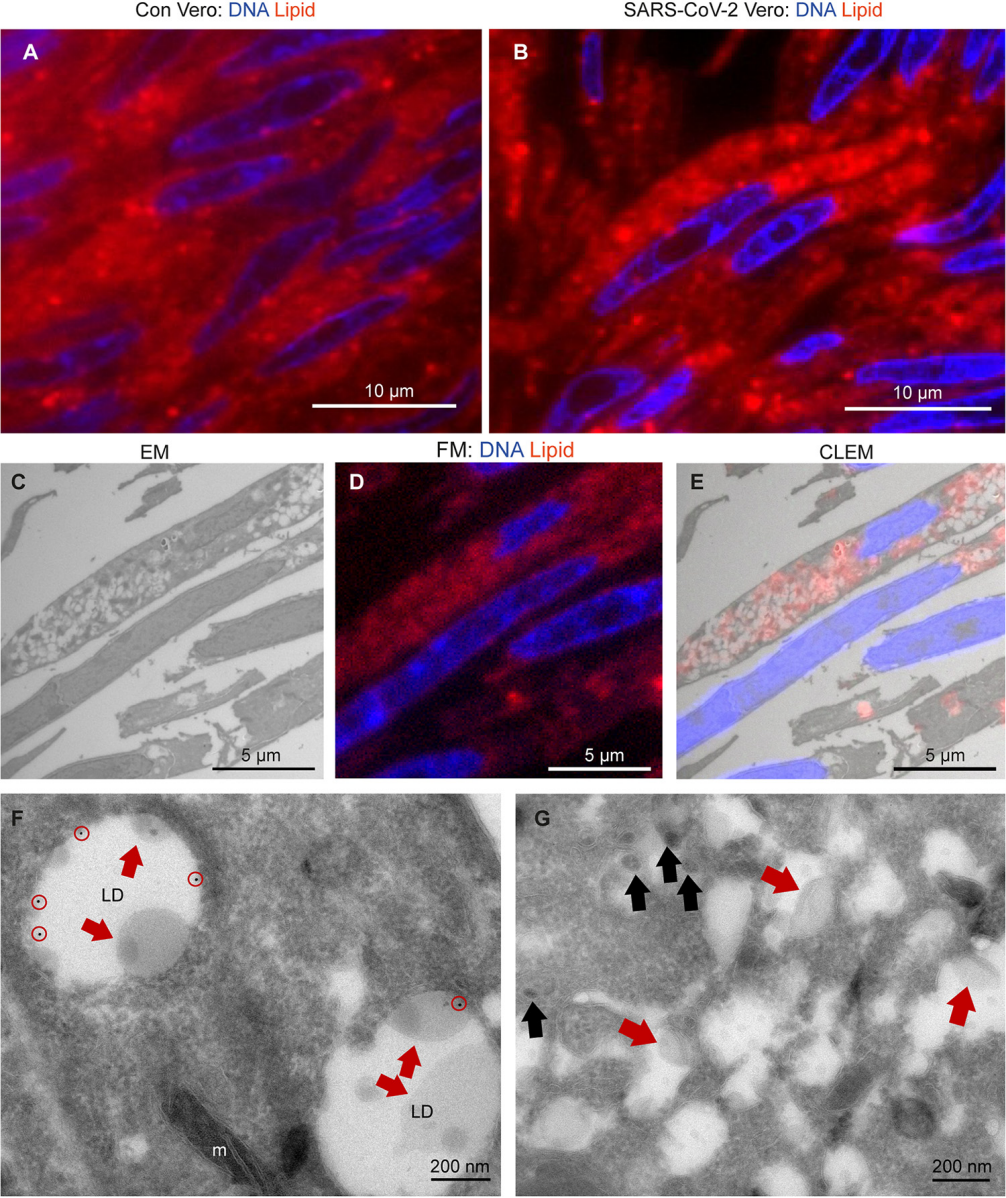
Lipid accumulates in e-lucent compartments more densely in infected Vero cells. Fluorescence microscopy of DNA and lipid staining with Nile red in (A) the uninfected control (Con) Vero cells and (B) cells infected with SARS-CoV-2 for 24 h. (C) Electron microscopy of infected cells. (D) Fluorescence microscopy of the same cells. (E) Correlative light-electron microscopy (CLEM) showing lipid staining at e-lucent compartments in the electron microscope. Immuno-EM labeling for lipid droplet marker perilipin-2 in (F) uninfected Vero cells and (G) cells infected with SARS-CoV-2 for 24 h. Blue color in panels A, B, D, and E shows the nuclei stained with Hoechst, and red shows the lipids stained with Nile red. In electron micrographs, lipid-like structure is denoted by red arrows, virus particles by black arrows, immunogold labeling of perilipin-2 by red circles, mitochondria by m, and lipid droplets by LD.

CD63 is also detected on early endosomes but is not present on the majority of the e-lucent structures detected in clusters in SARS-CoV-2-infected cells (Fig. S4). These structures seem to be induced by the virus infection, as uninfected cells contain larger lipid droplets but not the clustered e-lucent structures of 327 nm ± 130 nm. High magnification analyses reveal that the e-lucent compartments appear to be filled with lipid-like structures (Fig. 1, Fig. S1B, Fig. S4F and H), much like we described previously for Mycobacterium tuberculosis-infected cells (52). Therefore, Nile red staining was performed on both uninfected and SARS-CoV-2-infected Vero cells, and a clear increase in Nile red signal is observed in infected cells (Fig. 2). Indeed, others already demonstrated that lipid accumulation occurs after SARS-CoV-2 infection in Vero cells (53, 54). To prove that the e-lucent compartments detected with EM are Nile red positive and thus lipid-containing compartments, both FM and EM were performed on the same section and combined in a CLEM image (Fig. 2C to E). These CLEM images demonstrate that at least a part of the e-lucent compartments is lipid filled. The structure of these compartments is not identical to that of lipid droplets (LD), so we used an antibody specific for perilipin-2, which is known to localize in LD (55), to determine if the SARS-CoV-2-induced lipid-filled compartments are in fact lipid droplets. Immunogold labeling was present on typical LD in uninfected Vero cells but not on the lipid-filled compartments detected in SARS-CoV-2-infected cells (Fig. 2F and G). Based on the absence of both the lysosomal marker CD63 and LD marker perilipin-2, these e-lucent structures are not lysosomes or LD but rather novel lipid-filled compartments induced by SARS-CoV-2 infection.

### Localization of M-protein and nonstructural proteins nsp4 and nsp13.

**FIG 3:**
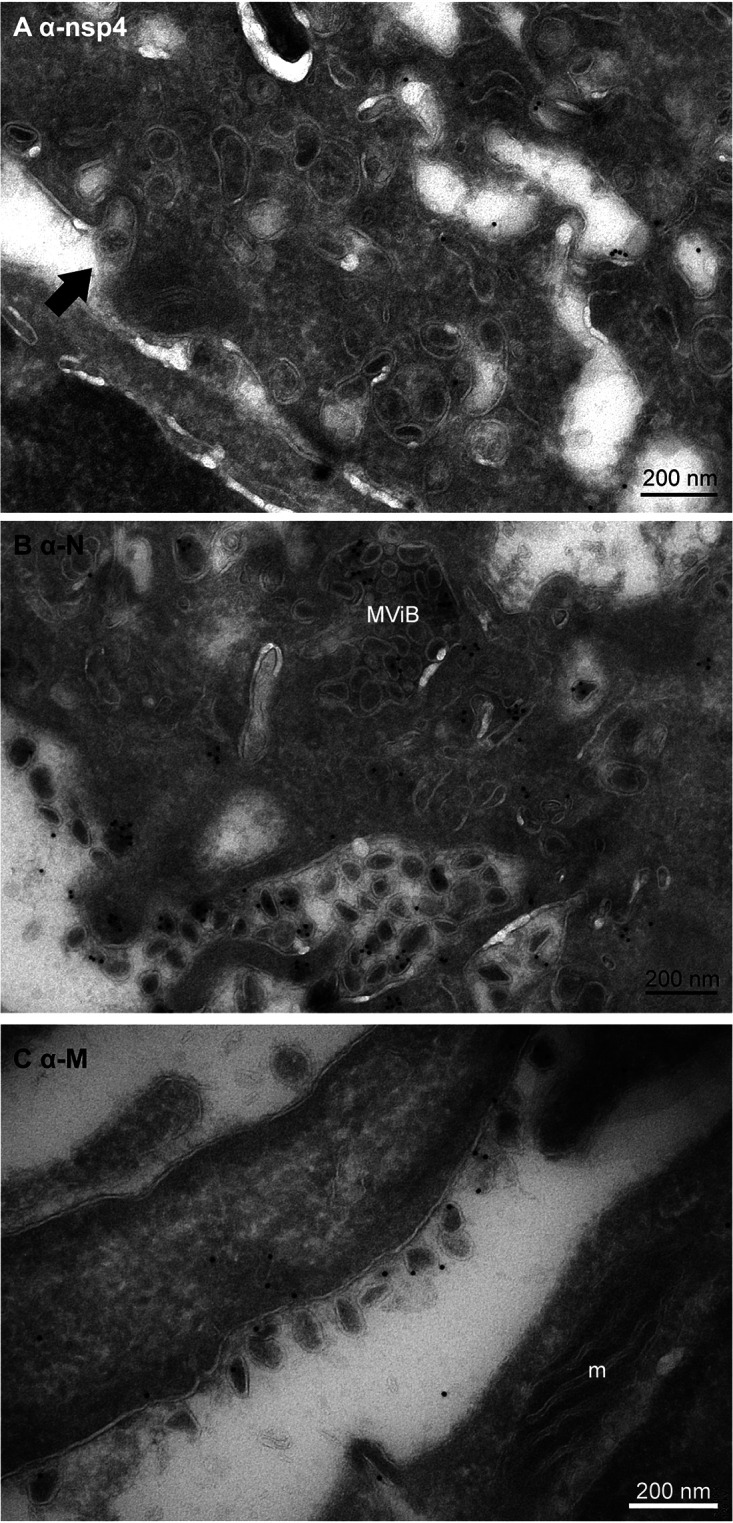
Release of virus particles from Vero cells infected with SARS-CoV-2 for 24 h. EM micrographs demonstrate (A) lack of immunogold labeling on extracellular virus particle using anti-nsp4, a nonstructural protein of SARS-CoV-2 (black arrow), (B) extracellular virus particles labeled with anti-N-protein, and (C) anti-M-protein also labeled on extracellular virus particles. Here, m represents mitochondrion, MViB multivirus body.

The localization of different viral proteins in cultured cells can be used to understand the pathology and replication of SARS-CoV-2 in lung tissue of COVID-19 patients. In infected Vero cells, the same procedures used for N-protein were applied to detect nsp3, but immunogold label is very limited, and thus, we conclude that this antibody does not recognize its substrate after 14 days of glutaraldehyde-paraformaldehyde fixation (Table 2). The nonstructural proteins nsp4 and nsp13 are detected on vesicles located nearby and attached to the Golgi stacks (Fig. S2). The signal of nsp13 is limited to a few gold particles per Golgi stack, and nsp4 is more distinct but also has some background on mitochondria (Fig. S4G). The M-protein abundantly labels Golgi stacks and vesicles around the Golgi. Interestingly, nsp4, nsp13, and M are also detected on MViBs (Fig. S3) and at e-lucent lipid-filled compartments, while uninfected cells are unlabeled (Fig. 1, Fig. S4). These structures resemble double-membrane vesicles (DMVs) or single-membrane vesicles described for MHV-, SARS-CoV-1-, SARS-CoV-2-, and MERS-CoV-infected cells (3, 5, 16–18, 23, 49). Single-membrane vesicles are proposed to be derived from the ER-to-Golgi intermediate compartment (56) and play a role in the secretion of virus to be released into extracellular space. With immuno-EM labeling only on some cellular compartments, a double membrane was detected (Fig. S4H, blue arrows), which could be explained by the EM technique used. Rather than performing high-pressure fixation and freeze substitution (3) or cryo-EM (17, 18), we used conventional fixation to be able to compare Vero cells with lung tissues of COVID-19 patients. It is possible that the double membranes are lost during fixation for immuno-EM, as Snijder et al. already demonstrated in 2006 (16). Another limitation of the immuno-EM is that no clear spike proteins are detected on extracellular virus particles based on the ultrastructure (immunogold labeling with antibodies specific for the SARS-CoV-2 spike protein were thus far not successful) (Fig. 3), though conventional sample preparation using osmium staining and embedding does show spikes (11, 40), as does cryo-EM (17, 18). Extracellular virus particles are immunolabeled for both N- and M-protein. Interestingly, the majority of the extracellular virus particles are not spherical but, rather, oval-shaped. The subcellular localization of N-protein, M-protein, and nsps in infected Vero cells is summarized in Table 2, and translation of this knowledge to patient material could be essential for understanding COVID-19 pathogenesis in patients. As immunolocalization with the antibodies against N-protein, M-protein, and nsp4 is specific and survives glutaraldehyde fixation, these antibodies can be used for analysis of lung tissues.

**TABLE 2:** Immunogold labeling of viral proteins in SARS-CoV-2-infected Vero cells^*a*^

Location in cell culture	Presence of protein:					
	N^*b*^	M	nsp3	nsp4	nsp13	CD63
Virus particle	+	+	−	−	−	−
Golgi	+/−	+	−	+	+/−	−
MViB	+	+	−	+/−	+/−	+/−
MLB	−	−	−	−	−	+
E-lucent compartment	+/−	+/−	−	+	+/−	−
Extracellular virus particle	+	+	−	−	−	−

a Presence of immunogold labeling on virus particles, Golgi, multivirus bodies (MViB), multilamellar bodies (MLB), e-lucent compartments, and extracellular virus particles in Vero cells infected with SARS-CoV-2 for 24 h.

b +, present; −, absent; +/−, present but less prominent.

### Immuno-EM on lung of COVID-19 patients.

**FIG 4:**
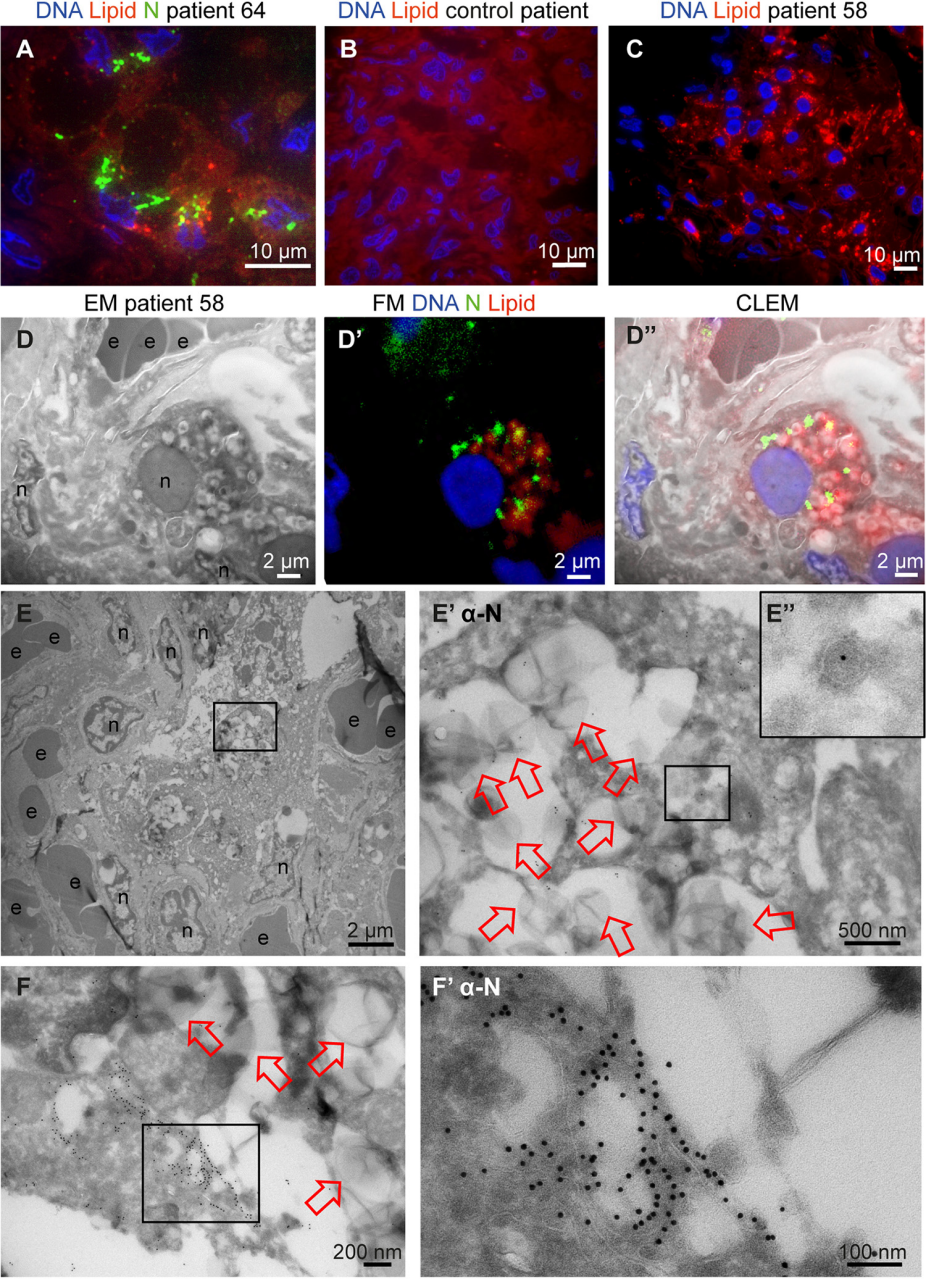
N-protein in e-lucent compartments in lung COVID-19 patient. Lung from control and infected patients was sectioned semithin for FM (A to C) or CLEM (D) and stained with Hoechst (blue) to identify nuclei, Nile red (red) to denote lipid, or anti-N-protein (green) to show N-protein, or it was ultrathin-sectioned for EM (E and F) and immunogold labeled using anti-N-protein followed by secondary antibody tagged with 10-nm gold particles. (A) COVID-19-infected lung showing accumulations of N-protein and Nile red stained lipids. (B) Overview of an uninfected control lung with no N-protein or lipid accumulation. (C) Overview of infected lung with lipid accumulation. (D) Identical section analysed by CLEM of infected lung demonstrate by EM e-lucent compartments (D) by FM Nile red and N-protein labelling (D’) and by the overlay of the FM on the EM micrograph the presence of Nile Red and N-protein on e-lucent compartments (D’’). Immunogold labeling of infected lung with antibody against N-protein at low magnification (E) and magnified region from boxed area where lipid-like structures (open red arrows) are visible (E’) and a single virus particle with N labeling (E’’). (F) Low magnification of N-protein labeling on membrane structures near the e-lucent compartments. (F’) high magnification of clusters of N-protein labeling. Erythrocytes represented by e, nucleus by n, open red arrow lipid-like structures, and boxed areas enlarged region.

**FIG 5:**
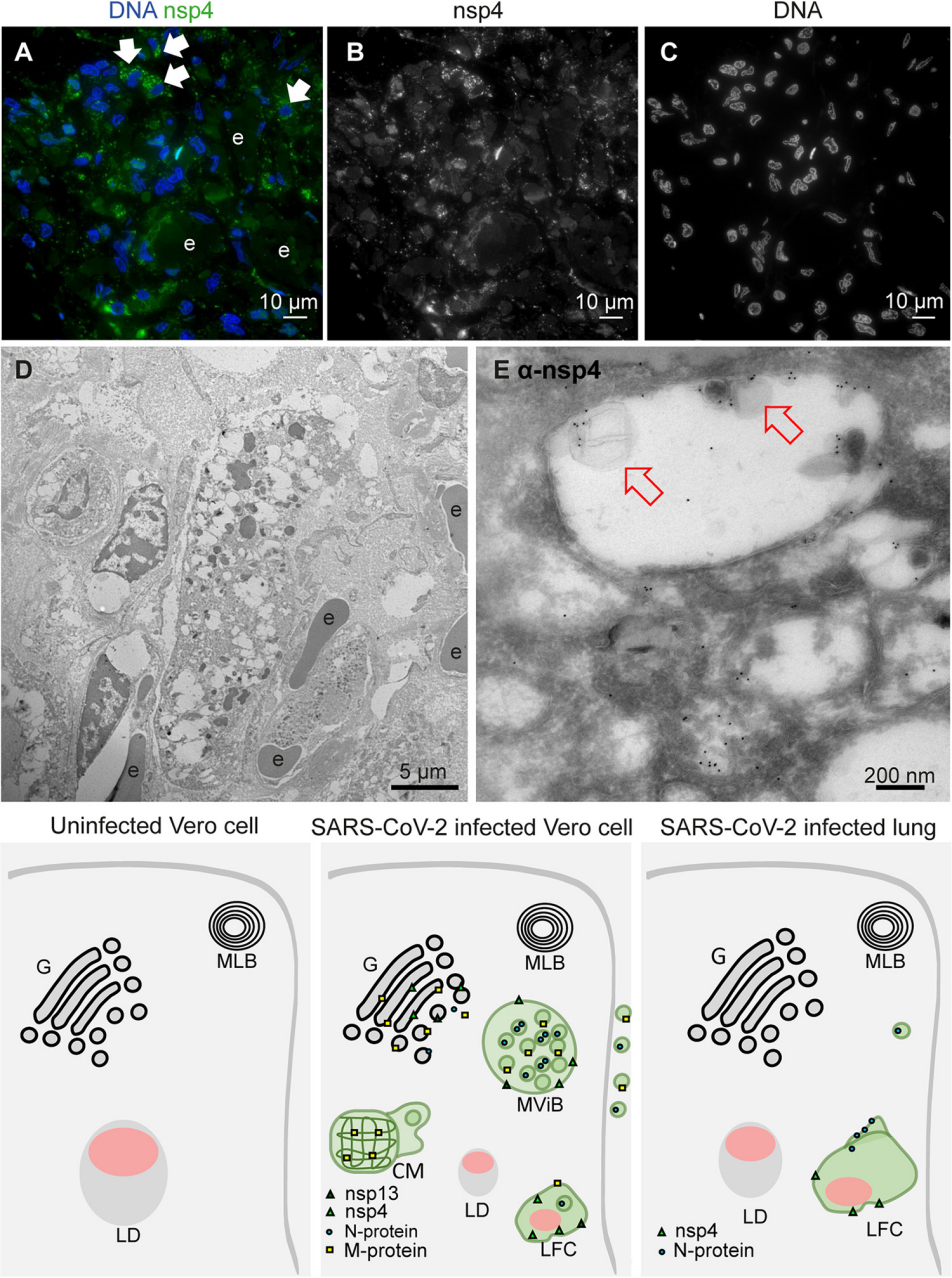
Nonstructural protein 4 in e-lucent compartments of infected lung. Lung tissue of COVID-19 patient 58 was sectioned semithin for FM with (A) nuclei, stained with Hoechst (blue), nsp4 stained with Alexa 488 (green) in nsp4 positive cells indicated by white arrows and in black and white, and erythrocytes represented by e. Separate channels of nsp4 (B) and DNA (C). Ultrathin sections of infected lung immunogold labeled against nsp4 and 10-nm gold particles in overview (D) and at higher magnification (E). E-lucent compartments with nsp4 labeling on membrane and lipid-like structures (open red arrows); erythrocytes represented by e. (F) Schematic representation of uninfected Vero cells, SARS-CoV-2-infected Vero cells, and lung tissue of COVID-19 patient summarizing presence cellular organelles and subcellular localization viral proteins. In black, host compartments; in green, viral compartments; in pink, lipid-like structures; CM, convoluted membrane; G, Golgi; LD, lipid droplet; MLB, multilamellar bodies; MViB, multivirus body; LFC, lipid-filled compartment and immunolabeling viral proteins. Dark green triangle, nsp13; light green triangle, nsp4; blue circle, N-protein; yellow square, M-protein.

In lung of COVID-19 patients, we searched for the presence of virus and replication organelles using antibodies selected on infected Vero cells. Materials of 7 COVID-19 patients from a prospective autopsy cohort study performed at Amsterdam University Medical Centers (UMC) (24) were included. With informed consent from relatives, full body autopsies were performed, and lung material was fixed for EM analysis. Materials were fixed for 1, 3, or 14 days. From those 7 patients, the lung tissues of 2 were too damaged to use for EM due to a postmortem delay. From our previous light microscopy analysis (24), we learned that only in a part of the lung tissue of a COVID-19 patient can N-protein be detected, and virus particles are difficult to find. Thus, in order to find the infected region of interest (ROI), we first performed fluorescence microscopy on sections of tissues processed for EM, so that when we identified an ROI containing viral proteins, EM could be performed (approached as in van Leeuwen et al. [57]). Semithick 0.3-μm slices were incubated with antibodies against SARS-CoV-1 nsp3, nsp4, and nsp13 and structural proteins N-, M-, and S-protein. We focused on areas near small blood vessels and alveolar walls, as our previous light microscopy (LM) analysis revealed infected cells present along the alveolar walls. These cells were identified to be pneumocytes, stromal cells in the septa, endothelial cells in the septal capillaries, and alveolar macrophages (24). Fluorescence microscopy showed that the N-protein (Fig. 4) and nsp4 (Fig. 5) could be detected. Noteworthy is the higher background for the M antibody and the relatively low labeling for nsp3 and nsp13 (Table 3).

**TABLE 3:** Immunogold labeling of viral proteins in SARS-CoV-2-infected lung^*a*^

Location in lung	Presence of protein:					
	N^*b*^	M	nsp3	nsp4	nsp13	CD63
Virus particle	+/−	−	−	−	−	−
Golgi	−	−	−	−	−	−
MViB	−	−	−	−	−	−
MLB	−	−	−	−	−	+
E-lucent compartment	+/−	+/−	−	+	−	−
Extracellular virus particle	−	−	−	−	−	−

a Presence of immunogold labeling on virus particles, Golgi, multivirus bodies (MViB), multilamellar bodies (MLB), e-lucent compartments, and extracellular virus particles in patient 58 and 64 infected with SARS-CoV-2.

b +, present; −, absent; +/−, present but less prominent.

Thus, in lung tissue from COVID-19 patients, an ROI was selected by FM using the N-protein antibody. In one patient (patient 64), relatively large clusters of N-protein were detected (Fig. 4A) often in a perinuclear region. As in Vero cells (Fig. 2), an increase in lipid accumulation was observed (Fig. 4B and C). Nile red staining was combined with N-protein labeling, and N-protein and lipid accumulations localized in the same general areas but did not colocalize at the same subcellular localization. Control lung material processed identically to COVID-19 patient material and tested for lipid accumulation demonstrated homogeneous background staining. Sections of 150 nm were analyzed with both FM and EM and combined (Fig. 4D). In line with our CLEM data on the Vero cells, performing CLEM on lung tissue demonstrated that lung tissue also accumulates lipid in e-lucent compartments. Then, ultrathin 60-nm cryo-sections were cut, and protein A conjugated to 10-nm gold particles was used to label N-protein.

The ultrastructure of the lung tissue is reasonable given the fact that this is postmortem material and it is from a patient with COVID-19. The tissue is unlike healthy lung tissue, not ventilated but instead filled with erythrocytes and packed with inflammatory cells infiltrating the alveolar lumen and interalveolar septa. It is not always possible to identify the cell type, especially when the nucleus is not present in the 60-nm thin section. N-protein is detected in cells with large e-lucent compartments, with some label found in e-lucent, lipid-filled compartments. Only a few spherical single-membrane structures with N-protein were detected (Fig. 4’ inset). These might be virus-like particles, but due to the low labeling (1 gold particle), the on-average-larger diameter (110 nm), and an atypical localization in the cytosol, overinterpretation is possible. Nonetheless, large clusters of viruses were not detected. In addition to the limited labeling on small round vesicles, N-protein was also present on membranous structures close to e-lucent compartments (Fig. 4F, Fig. S5). These structures were not present in all patients; from the 7 patients investigated, 2 had clusters of proteins detectable with the SARS-CoV-1 anti-N-protein. In patient 64 (patient description in reference 24), relatively large N-protein clusters at the e-lucent compartments were detected (Fig. 4F, Fig. S5A to D), and smaller clusters were detected in patient 58, albeit at a similar location (on membrane clusters near the e-lucent compartments [Fig. S5E and F]).

Using FM, nsp4 was identified in the same ROI of lung tissue used for detection of N-protein (Fig. 5). Cells positive for nsp4 were present in various tissue compartments. Although background labeling was detected, some cells were brightly positive. Immuno-EM demonstrated nsp4 on e-lucent compartments, which were filled with lipid-like structures. A small amount of label was detected on mitochondria, which should be regarded as background labeling, as this was also present on uninfected Vero cells (Fig. S4G). The summary of subcellular viral protein localization in lung is presented in Table 3 and, compared to the quantity of labeling in Vero cells, less labeling was detected in only limited compartments. The lipid-filled compartments, however, were positive for nsp4, and N-protein was accumulated close to these compartments. Like in Vero cells, lysosomal marker CD63 was absent from these compartments, and thus the lipid-filled compartments in lung were nonlysosomal. To our knowledge, these lipid-filled compartments, containing viral proteins nsp4 and N-protein, have not been identified before and need to be characterized further.

## DISCUSSION

Since the outbreak commenced, the identification of coronaviruses in lung by EM has been debated, and several articles had to be revised (46, 58, 59). Experienced electron microscopists (6) have summarized these studies and suggest using one of 3 strategies: (i) visualization of viral morphogenesis, (ii) immuno-EM or *in situ* hybridization, or (iii) visualization of particles *in situ* in tissue combined with biochemical evidence of viral presence. We chose immuno-EM with gold labeling using already validated antibodies raised against SARS-CoV-1 (49). Immuno-EM on Vero cells identified the monoclonal anti-SARS-CoV-1-N 46-4 to be the best for the detection of nucleocapsid N-protein. Virus particles were detected in the process of development as denoted by clusters of cytosolic N-protein surrounded by double membranes (Fig. 1, Fig. S1). Spherical and/or oval virus particles were detected in MViBs and in membrane clusters in the cell. The spherical virus particles were stable in size (87 ± 17 nm), and the oval-shaped virus particles were slightly larger (108 ± 27 in MViBs and 113 ± 28 nm for cytoplasmic) than the spherical ones, albeit these variances are not statistically different. It should be noted that in immuno-EM and at 24 h of infection, 20% of the virus particles were scored as oval. The functional difference between spherical versus oval-shaped virus particles still has to be discovered, but others have demonstrated that the oval or ellipsoidal-shaped virus particles contain more complexes of RNA and N-protein (42).

In lung of patients who had a fatal COVID-19 infection, virus-like particles were rarely detected even though the N-protein was detected in close proximity of the viral induced lipid-filled compartments. In Vero cells, however, N-protein was detected inside virus particles. It is possible that the difference is caused by incomplete fixation of lung or that ultrastructure is deteriorated in postmortem material. The overall ultrastructure of the tissue, however, is acceptable (Fig. 4 and 5), because the postmortem time was kept to a minimum and lung tissue was fixed within a few hours, during the first wave of COVID-19 infections in the Netherlands. Finally, it is important to note that the magnification of EM makes finding 90-nm virus particles in a tissue block of 1 by 1 mm^2^ extremely difficult. Still, some studies have detected an occasional cell filled with virus-like particles (43–46).

Interestingly, our CLEM data (Fig. 2 and 4) demonstrated that part of the e-lucent compartments we have detected in Vero cells and in lung of COVID-19 patients are lipid filled. Lipids are notoriously difficult to fix with glutaraldehyde and paraformaldehyde alone (60), and thus part of the compartments might have lost the lipid content, but lipid accumulation in virus-induced compartments is extremely interesting. For viruses of the *Flaviviridae* family, such as the dengue virus, hepatitis C virus, and others, lipid accumulation has been shown to be involved in viral replication (61–68). High-resolution EM studies on cryopreserved MHV-infected cells suggest DMVs to be filled with viral RNA with LD lying next to the DMVs (18). Also, in infected human pulmonary epithelial Calu-3 cells (13), lipid droplets are detected close to the DMVs. Fluorescence microscopy studies have demonstrated lipid accumulations in SARS-CoV-2-infection in Vero cells (53, 54), demonstrated that lipid accumulation is specific for SARS-CoV-2 and not for SARS-CoV-1 in a comparative electron microscopy study, and established an increase of LD in lungs from deceased COVID-19 patients. Here, using immuno-EM, combined with fluorescence microscopy, we demonstrate an induction of lipid-filled compartments and propose that the SARS-CoV-2 infection-induced compartments are not LDs, as they are irregular in shape and have a morphology different from that of spherical perilipin-2-stained LDs. Also, the clearly visible membrane (Fig. S2), containing transmembrane proteins nsp4 and nsp13, demonstrates that the virus-induced lipid-filled compartments are surrounded by a bilayer, while lipid droplets are surrounded by a monolayer of phospholipids. Taken together, SARS-CoV-2 infection induces novel lipid-filled compartments, different from LD or endosomes but with viral proteins nsp4 and N-protein.

Here, we used the SARS-CoV-2-infected Vero cells to determine in which fixation conditions and on which structures we could detect viral proteins to compare those with lung tissues from patients conserved under the same conditions. We noticed that not all structures present in Vero cells can be detected at the last stage of infection. A virus-induced structure that is well described is convoluted membranes, which was detected in Vero cells (Fig. 1B) but not in lung at the final state of infection. In addition, multivirus bodies were detected specifically in Vero cells and not in lung (Fig. 1, Fig. S1 and S3). The MViBs are different from lysosomal MVBs, based on the fact that the MViBs are not CD63 positive and based on the size, morphology, and M-, N-protein labeling detected within the structures. In the lung of patients with fatal COVID-19, no MViBs were detected. Also, double-membrane vesicles (DMVs) have been described in several EM studies (2, 3, 5, 15–18) but are not so obvious in our immuno-EM images; only a few double membranes were identified surrounding e-lucent compartments (Fig. S4, blue arrows), possibly due to fixation limitations, as shown before by Snijder et al. (16). As double membranes were not recognizable, DMVs were not annotated in this study. Recent comparison of SARS-CoV-2-infected Vero cells versus lung organoids demonstrated that the subcellular trafficking in Vero cells might be different (69), which can explain the presence of MViB in Vero and absence of these organelles in lung. Also, the infections stage could be an explanation, as we have analyzed postmortem material and thus the last stage of the disease.

Remarkably, N-protein and nsp4 are detected in lung of patients in the last stage of the disease. It seems unlikely that only these 2 proteins are still produced by active replication of the virus but, rather, likely that both N-protein and nsp4 are more stable proteins and thus not degraded. The gene encoding the N-protein is conserved and stable, and the N-protein itself is both highly immunogenic and highly expressed during infection (70). Work on patients with a SARS-CoV-1 infection demonstrated elevated levels of IgG antibodies against N-protein (71) and showed that N-protein is an antigen for T-cell responses, inducing SARS-CoV-1-specific T-cell proliferation and cytotoxic activity (72–74). Also, in an increasing number of case studies, anti-N IgGs were detected in patients with severe COVID-19 (75), and in children, 5 out of 6 produced neutralizing IgG and IgM antibodies targeted to the N- and S-proteins of SARS-CoV-2 (76). Interestingly, recent reports show that immune responses to the N-protein have been associated with poor clinical outcomes (77) and correlate with severity of COVID-19 (78).

In the current electron microscopy study, we detected in fatal COVID-19 infections using SARS-CoV-specific antibodies the stable presence of N-protein and nsp4 on novel lipid-filled compartments. Already, it has been demonstrated that pharmacological inhibition via a key enzyme for LD formation affected SARS-CoV2 replication in cells (51), suggesting that lipid accumulation is a potential drug target. The identification of the lipid-filled compartments could serve as a hallmark for SARS-CoV-2 infections, especially since finding virus particles is challenging. It is possible that lipid-filled viral protein-containing compartments play a role in the secondary effects of the disease. The uncontrolled immune responses causing the devastating damage of COVID-19 could be a response to the proteins or even lipids accumulating in these novel subcellular compartments and thus should be further investigated.

## MATERIALS AND METHODS

### EM infection and fixation of cultured Vero cells.

Vero E6 cells were seeded (2.5 × 10^6^ cells/T75 flask) 1 day before infection in minimal essential medium (MEM)/25 mM HEPES/2% fetal calf serum with penicillin and streptomycin. Cells (∼5 × 10^6^ cells/T75) were infected with multiplicity of infection (MOI) of 0.2 by adding the virus (nCoV-2019/Melb-1 [4.3 × 10^6^ PFU/mL]) to each T75 flask. Incubation was performed at 37°C for 24 h. Then, cells with and, as a control, without virus were fixed in 1 part medium plus 1 part 6% paraformaldehyde (PFA) plus 0.4% glutaraldehyde (GA) in 0.4 M PHEM buffer (240 mM PIPES [piperazine-N,N′-bis{2-ethanesulfonic acid}], 100 mM HEPES, 8 mM MgCl_2_, and 40 mM EGTA at pH 6.9). After 1, 3, and 14 days of fixation, samples were transferred to storage buffer (0.2 M PHEM with 0.5% PFA).

### Collection and initial fixation of tissue from COVID-19 patients.

Autopsies were performed at Amsterdam University Medical Centers (UMC), VU Medical Center, Academic Medical Center, the Netherlands, according to the declaration of Helsinki. For this EM study, 7 patients with clinically confirmed COVID-19 for whom autopsy was requested were included (Table 4). Ethical approval was granted by the institutional review board of Amsterdam UMC (METC 2020.167). As described previously (24), COVID-19 was confirmed by quantitative real-time reverse transcriptase PCR (RT-PCR), and informed consent was obtained from the decedents’ next of kin. During autopsy, lungs for conventional EM were fixed in Karnovski fixative with 4% PFA and 1% GA in 0.1 M sodium cacodylate buffer. To avoid safety problems, samples were fixed for 14 days and transferred to storage buffer or embedded in gelatin and snap-frozen.

**TABLE 4:** Patient description^*a*^

Patient	Infection stadium	Sex	Age	COV-N^*b*^	Remarks
SVU 20-58	Limited infected cells in lung, limited systemic presence (HPB tract)	F	72	+	Data presented
SVU 20-39	Severe infected cells in lungs, systemic presence (GI tract)	M	73	+	
SVU 20-63	No presence in lung, limited presence in the heart	M	74	+	
SVU 20-64	Limited presence in the lung, no systemic presence	F	68	+	Data presented
SVU 20-155		F	75	+	
SVU 20-163		M	61	+	
SVU 20-174		M	78	+	
SVU 20-129	Control noncovid	M	68	−	
T18-5683	Control noncovid	F	5	−	Data presented
T18-10645	Control noncovid	F	15	−	

a Information of patients from who autopsy material was taken with informed consent and fixed for electron microscopy. In this study, electron micrographs were used from patients SVU 20–58, SVU 20–64, and control T18-5683.

b COV-N represents SARS-CoV-1-N protein detection.

### Embedding and sectioning.

After fixation, cells and tissue were washed 3 times with phosphate-buffered saline (PBS) plus 0.02 M glycine (Merck, K27662101) to remove fixative. Cells were pelleted by centrifugation at 980 × *g* for 3 min. Supernatant was removed, cells were directly embedded in 12% gelatin (Sigma, G2500-500G) in 0.1 M phosphate buffer and pelleted by centrifugation for 3 min at 10,950 × *g* and solidified on ice, and blocks of ∼1 mm^2^ were cut with a razor blade. Lung tissue was cut into blocks of 1 to 2 mm^2^ and imbedded in a gelatin series of 2%, 6%, and 12% gelatin in 0.1 M phosphate buffer. Blocks of cells or tissue were incubated overnight in 2.3 M sucrose at 4°C (Merck, K17687153) in 0.1 M phosphate buffer. Then, samples were snap-frozen and stored in liquid nitrogen. Sectioning was performed using a diamond knife (Diatome cryo-immuno) on a Leica Ultracut UC6 cryo-ultramicrotome. Semithin sections (150 to 300 nm) were made at −80°C, and ultrathin sections were made at −120°C. The sections were transferred to a Formvar-coated copper grid, gold finder grid, or glass slide in a droplet of 1 part 2% methylcellulose (Sigma, M6385-250G) to 1 part 2.3 M sucrose. Sections were stored at 4°C until labeling.

### Immunofluorescence labeling.

Semithin cryo-sections were transferred to gold finder grids for EM or to glass slides for light microscopy (LM) and washed with PBS plus 0.02 M glycine. Then, for LM, semithin sections were incubated on primary antibody for 1 h in PBS plus 0.1% bovine serum albumin (Sigma, A4503-50G) and washed with PBS plus 0.02 M glycine. Thereafter, they were incubated with secondary antibody conjugated to Alexa 488 (Mol. Probes, A32731), and in the last 5 min, Nile red (Sigma, 72485) and Hoechst 33342 (Thermo Fisher, H3570) were added. After washing with PBS, a cover slip was mounted with Vectashield (Vector laboratories, H-1000). Glass slides were imaged using a Leica DM6 widefield microscope with a 100× oil objective. Images were analyzed using ImageJ FIJI.

### Immunogold labeling.

For EM, ultrathin sections were picked up and placed on 150 mesh copper grids and incubated on 2% gelatin in 0.1 M phosphate buffer for 30 min at 37°C. Then, at room temperature, grids were washed with PBS plus 0.02 M glycine and blocked with 1% bovine serum albumin (BSA) in PBS. Grids were incubated with primary antibody in 1% BSA in PBS for 45 min. Then, grids were washed with PBS plus 0.02 M glycine. When the primary antibody was an unlabeled mouse monoclonal antibody, a secondary antibody, raised against mouse serum, was used as a bridge to enhance labeling, followed by incubation with protein A conjugated with colloidal gold. In this case, background blocking was done by 0.1% BSA in PBS plus 0.02 M glycine, followed by incubation on rabbit anti-mouse antibody (Z0259, Dako) for 20 min and washed with PBS plus 0.02 M glycine. Again, grids were incubated in blocking solution and subsequently with protein A conjugated to 10-nm gold (Utrecht University). After washing with PBS, grids were incubated with 1% glutaraldehyde in PBS to fix the antibody-gold complex and washed 10 times for 2 min each with water. To contrast the samples, grids were incubated with uranyl acetate in 2% methylcellulose for 5 min, and the excess liquid was blotted from the grids with filter paper. Grids were imaged using an FEI Tecnai 120 kV transmission electron microscope with a Veleta or Xarosa camera (EMSIS). Images were analyzed using ImageJ FIJI.

### Correlative light and electron microscopy.

For CLEM, we used a method described earlier (57). In short, grids were washed with PBS plus 0.02 M glycine, incubated for 1 h with primary antibody, and again washed with PBS plus 0.02 M glycine. Thereafter, grids were incubated with secondary antibody Alexa 488, and in the last 5 min, Nile red (Sigma, 72485) and Hoechst 33342 (Thermo Fisher, H3570) were added. After washing in PBS, the grids were mounted in between a glass slide and a coverslip in a droplet of Vectashield. CLEM samples were imaged on a Leica DM6 widefield microscope using a 100× oil objective. Images were analyzed using LasX. After widefield imaging, the coverslip was removed from the glass slide by pipetting PBS in between the coverslip and the glass slide. Vectashield was removed by washing the grid with MilliQ water at 37°C. Thereafter, the grids were contrasted and imaged as described above. The correlation was performed using ICY eC-CLEM software. A list of materials is provided in Table 5.

**TABLE 5:** List of materials

Reagent or resource	Source	Identifier
Antibodies		
Rabbit polyclonal anti-nsp3	Kind gift from Snijder laboratory	Leiden University Medical Centre (16)
Rabbit polyclonal anti-nsp4	Kind gift from Snijder laboratory	Leiden University Medical Centre (77)
Rabbit polyclonal anti-nsp13	Kind gift from Snijder laboratory	Leiden University Medical Centre (16)
Rabbit polyclonal anti-M	Kind gift from Snijder laboratory	Leiden University Medical Centre (16)
Rabbit polyclonal anti-N anti-SARS-CoV-1-N (46-4)	Kind gift from Snijder laboratory	Leiden University Medical Centre (79)
Rabbit polyclonal anti-N	Sino Biological Inc	Cat. no. 40143-T62
Monoclonal mouse recombinant SARS-CoV anti-N	Sino Biological Inc	Cat. no. 40143-MM05
Mouse monoclonal anti-CD63	Santa Cruz	Cat. no. MX49.129.5; RRIDl1817
Mouse monoclonal anti-perilipin-2	Progen	Cat. no. 610102; RRID00300-05
Rabbit Bridging anti-mouse	DAKO	Cat. no. Z0259; RRID20007985
Goat anti-mouse Alexa488	Life technologies	Cat. no. A21242; RRID1345066
Goat anti-rabbit Alexa488	Mol. Probes, Invitrogen	Cat. no. A27034; RRID2031072
Protein A conjugated to 10-nm gold	Utrecht University	https://cellbiology-utrecht.nl/products.html
Chemicals		
Nile red	Sigma-Aldrich	72485
Hoechst 33342	Thermo Fisher	H3570
Phosphate-buffered saline	Gibco	18912-014
Glycine	Merck	K27662101
Gelatin	Sigma-Aldrich	G2500-500G; CAS9000-70-8
Methylcellulose	Sigma-Aldrich	M6385-250G; CAS9004-67-5
Bovine serum albumin	Sigma-Aldrich	A4503-50G; CAS9048-46-8
Vectashield	Vector Laboratories	H-1000
Uranyl acetate	EMS	22400
Virus strain		
nCoV-2019/Melb-1	Kind gift from Snijder laboratory	
Experimental models: cell line		
Vero cells	Kind gift from Snijder laboratory	
